# Novel biomarkers and neuroimaging techniques in Parkinson’s disease

**DOI:** 10.1007/s00415-024-12187-6

**Published:** 2024-01-17

**Authors:** A. Saw, S. Bari, Neil P. Robertson

**Affiliations:** 1 Department of Neurology, University Hospital of Wales, Heath Park, Cardiff, CF14 4XN UK; 2 Institute of Psychological Medicine and Clinical Neuroscience, Cardiff University, University Hospital of Wales, Heath Park, Cardiff, CF14 4XN UK

Parkinson’s disease (PD) is one of the world’s most common neurodegenerative disorders, with global prevalence now expected to increase to 14 million in 2040. Although PD remains a leading cause of neurological disability, there remain a number of hurdles to optimise long-term clinical management. In particular, PD is currently diagnosed by clinical criteria which heavily rely on the expression of classical motor symptoms which occur relatively late in the pathological process. However, earlier or pre-symptomatic diagnosis would offer the opportunity to explore the role of neuroprotective or disease-modifying therapies. In addition, a more detailed understanding of the mechanisms of non-pharmacological interventions would allow the application of more personalised approach to interventions and a better understanding of underlying disease processes. In this month’s journal club, we review a study using cerebrospinal fluid (CSF) α-synuclein as novel diagnostic biomarker and two neuroimaging studies exploring the neural mechanisms responsible for success of aerobic exercise and optimisation of DBS parameters in PD.

## Assessment of heterogeneity among participants in the Parkinson’s Progression Markers Initiative cohort using α-synuclein seed amplification: a cross-sectional study

This is a cross-sectional observational study designed to assess diagnostic performance of CSF α-synuclein seed amplification assays (SAA) for early identification of people at risk of PD and detection of disease heterogeneity. 1123 participants were identified from 33 global outpatient neurology practices over a period of 9 years. 545 had PD, 51 were prodromal participants with either rapid eye movement sleep behaviour disorder (RBD) or hyposmia, and 54 had Parkinsonism but without imaging evidence of dopaminergic deficit (SWEDD). 310 were non-manifesting carriers of LRRK2 and GBA variants and 163 were healthy controls. CSF samples were collected during enrolment for the Parkinson’s Progression Markers Initiative (PPMI) cohort. SAA positivity was determined by highest raw florescence in three technical replicates using a probabilistic algorithm.

CSF α-synuclein SAA was able to detect 87.7% of both sporadic and genetic PD cases (95% CI 84.9–90.5) whilst maintaining specificity of 96.3% (93.4–99.2) and was also able to differentiate healthy controls from PD cases. Moreover, there was diversity in the occurrence of positive α-synuclein SAA among different participant subgroups based on genetic and clinical features. Among genetic subgroups, GBA-positive PD displayed the highest positive rate (95.9%, 95%CI 90.4–100.0), followed by sporadic PD (93.3%, 09.8–95.8) and LRRK2 PD (67.5%, 59.2–75.8), respectively. Hyposmia was the most robust clinical predictor of a positive result with a 97.2% sensitivity (95.5–98.8). No significant associations were observed between autonomic function, cognitive test results, depression scores or RBD scores, and α-synuclein SAA status. Positive rate was highest for those with sporadic PD and typical olfactory deficit (98.6%) and lowest for those participants carrying the LRRK2 variant and normal olfaction (34.7%). Among prodromal groups, 44(86%) of 51 participants with RBD or hyposmia had positive α-synuclein SAA, but only 25(8%) of 310 non-manifesting genetic variant carriers were positive.

*Comment*: This study demonstrates that α-synuclein SAA can provide replicable and reliable information on identifying at-risk individuals from PD at an early stage of disease, with variability among genetic and clinical subgroups. It may offer opportunities for early targeted therapies in GBA, LRRK2 and α-synuclein-related PD. However, further clinical trials with longitudinal data and robust study designs are required to establish generalisability and clinical application.

Siderowf et al. Lancet Neuro. 2023 May; 22(5): 407–417.

## Aerobic exercise alters brain function and structure in Parkinson’s disease: a randomized controlled trial

Recently two large clinical trials (park-in-shape trial and SPARX trial) have provided convincing evidence that exercise can alleviate motor symptoms in PD. However, the mechanism underlying this effect is unclear. Animal models of PD have shown that aerobic exercise is able to stimulate protective and restorative forms of neuroplasticity in the sensorimotor network. In this study, functional MRI and diffusion-weighted imaging were used to investigate the systems-level cerebral changes associated with aerobic exercise in PD compared to an active control condition.

The Park-In-Shape trial is a single centre, prospective, double-blind, randomized, placebo-controlled trial evaluating the effect of aerobic exercise versus an active control (stretching) on symptom progression in PD. An unselected subset of 57 patients from the park-in-shape trial was divided in to two groups, aerobic exercise (25) and stretching (31). These patients underwent resting-state functional and structural magnetic resonance imaging (MRI) and an oculomotor cognitive control task (pro- and anti-saccades) at baseline and at 6-month follow-up.

The aerobic exercise group demonstrated a significantly larger posterior-to-anterior shift in cortico-striatal sensorimotor connectivity compared to the stretching group. Functional connectivity of the anterior putamen, relative to posterior putamen, increased in four clusters in the right primary motor cortex, primary somatosensory cortex, and premotor cortex. The aerobic exercise group had significantly lower change in global percentage-based volume compared to the stretching group (*p* = 0.049, *η*2*p* = 0.87); reduced global percentage-based volumes in the stretching group were also significant (*p* = 0.001, *g* = 0.6). The aerobic exercise group had significantly greater changes in error rates in a cognitive control task, depending on the condition, compared to the stretching group (*p* = 0.046, *η*2*p* = 0.11).

*Comment*: MRI, clinical, and behavioural results suggest that aerobic exercise can stabilise motor progression and enhance cognitive performance in individuals with PD by stimulating functional and structural neuroplasticity in both motor and cognitive brain networks in PD.

Johansson et al. ANN NEUROL 2022; 91:203–216, https://doi.org/10.1002/ana.26291

## Predicting optimal deep brain stimulation parameters for Parkinson’s disease using functional MRI and machine learning

DBS for PD has become the standard of care for selected patient groups. However, its use requires complex technical training and many hours of clinical testing to provide an appropriate stimulation at the best location within the target region to achieve maximal clinical benefit. These processes require significant healthcare resources and place considerable burdens on patients, limiting more widespread application. Current cutting-edge neuroimaging techniques now allow us to understand the physiological effects of DBS on the activity of brain circuits. This study builds on previous studies describing safety and feasibility of MRI in DBS patients and uses fMRI to identify a reproducible pattern of brain response to optimal DBS stimulation. This was then used to predict optimal DBS settings on the basis of brain response patterns with a machine learning (ML) algorithm trained on two new datasets: a prior clinically defined stimulation-optimized PD patient group and a stimulation-naïve PD patient cohort.

Patients undergoing subthalamic nucleus (STN) DBS (*n* = 62) were primarily recruited. Patients with internal globous pallidus (GPi) DBS (*n* = 5), an alternative stimulation location, were also included to determine whether different PD-DBS targets could also contribute to the ML model. Each session was 6.5 min in duration and employed a 30 s DBS-ON/OFF cycling paradigm repeated six times in which unilateral left DBS stimulation was delivered at patient-specific, clinically defined optimal and non-optimal contacts or voltages. Blood-oxygen-level-dependent (BOLD) signal was extracted from 16 motor and non-motor regions of interests (ROIs) determined a priori based on existing PET and SPECT literature. Brain regions with a significant increase (hot colours, positive *t*-values, DBS-ON > OFF) and decrease (cool colours, negative t-value, DBS-ON < OFF) (*p* < 0.001, cluster size = 50) in BOLD response were identified. Optimal contact showed changes in BOLD response in the left (ipsilateral) motor cortex and thalamus, and right (contralateral) cerebellum. After DBS surgery, PD patients underwent fMRI with fully implanted and active DBS systems. Contacts or voltages were screened, and associated fMRI patterns fed into a machine learning model, which then categorised these as optimal or non-optimal. The machine learning model was built with a train dataset using linear discriminant analysis and fivefold cross-validation.

*Comment*: Functional MRI MAP from DBS for PD can be used as an objective clinical tool for DBS programming to achieve optimal clinical benefit without costly and lengthy clinical visits. Results from this trial suggest that DBS-induced fMRI brain signatures obtained through machine learning algorithms are associated with optimal clinical benefit in PD. In addition, these findings suggest potential benefits of autonomous closed-loop DBS programming for other neurological diseases treated with DBS.

Boutet et al., *Nat Commun* **12**, 3043 (2021). https://doi.org/10.1038/s41467-021-23311-9

